# Fracture distribution in electric scooter accidents: a nationwide observational cohort study of 1,874 fractures from the Swedish fracture register

**DOI:** 10.1186/s13018-024-04940-4

**Published:** 2024-07-30

**Authors:** Björn Hernefalk, Anders Brüggemann, Olof Wolf

**Affiliations:** Department of Surgical Sciences, Orthopaedics, Uppsala University, Uppsala, Sweden

**Keywords:** E-scooter, Fractures, Trauma, Fracture distribution, Swedish fracture register, Transportation

## Abstract

**Background and purpose:**

Electric scooters (e-scooters) have become increasingly popular as a mode of transportation in recent years. The impact of e-scooter accidents on the healthcare system and resulting orthopaedic injuries remains largely unknown. This study describes the distribution of fractures caused by e-scooter accidents.

**Methods:**

All patients who had one or more fractures from e-scooter accidents registered in the Swedish Fracture Register (SFR) between 7 April 2019 and 30 December 2022 were included. Fractures were classified using the AO Foundation/Orthopaedic Trauma Association (AO/OTA) fracture classification system. We analysed the distribution of fractures, the proportion that required surgical management and seasonal variation of injuries.

**Results:**

During the study period, 1,874 fractures in 1,716 patients were registered in the SFR. The mean age of patients was 29 (SD 14) years and 70% of fractures occurred in males. High-energy accidents accounted for 299 fractures (16%). The most common fractures were of the hand (*n* = 363, 19%), wrist (*n* = 352, 19%) and proximal forearm (*n* = 356, 19%). Wrist fractures were the most common injury in children (*n* = 183), accounting for 44% of paediatric fractures. Surgical treatment was performed on 556 (30%) fractures, with wrist fractures being the most commonly treated in both adults (*n* = 78, 17%) and children (*n* = 36, 36%).

**Interpretation:**

Fractures caused by e-scooter accidents predominantly occur in the upper extremity. E-scooter accidents comprise a new source of injury requiring attention and surgical resources from an already strained healthcare system.

## Introduction

### Background

In recent years the popularity of electric scooters (e-scooters) has dramatically increased, providing a convenient alternative for short-distance travel. However, along with their growing popularity, there has been a noticeable increase in orthopaedic injuries. These injuries encompass a wide range of musculoskeletal trauma, most commonly affecting young males and resulting in upper extremity fractures [1–3]. This new type of transport injury have been shown to significantly affect health care costs [4].

E-scooter-related injuries can be caused by several factors. There is an inherent risk of riding a two-wheeled vehicle with disproportionately small wheels and a short wheelbase. Rider inexperience, lack of protective gear and intoxication could further increase the risk of sustaining a fracture, along with environmental factors and traffic conditions.

In order to reduce the number of accidents and develop effective prevention strategies, it is important for health-care professionals, policymakers and the general public to gain further knowledge regarding the nature of e-scooter related injuries.

### Objective

This study aimed to explore types of fracture, age and sex distribution in individuals who sustained fractures from e-scooter accidents. A further aim was to study the proportion of surgically treated patients and the seasonal variation in e-scooter accidents.

## Patients and methods

### Study design and setting

This observational study was based on data from the Swedish Fracture Register (SFR), which contains data on injury mechanism, fracture classification and treatment (surgical and non-surgical) of Swedish citizens with a fracture sustained in Sweden [5]. The injury mechanism is registered by the treating orthopaedic surgeon. Since the introduction of e-scooters, this mode of transportation has been added as a subtype to the registration of bicycle accidents. Fractures are classified according to the AO/OTA classification (2007 version) [6]. Several studies have investigated the accuracy of the SFR classification and found it reliable [7–9]. The coverage of the SFR has gradually increased from one active department in 2011 to 75% coverage in 2016 and full national coverage in 2021 (54 of 54 orthopaedic departments).

A comparison with the National Patient Register (NPR) in 2022 demonstrated a completeness of the SFR of approximately 60% for all fractures and 81% for femoral fractures. These figures are conservative as the NPR overestimates the number of fractures, mostly due to miscoding old fractures as new at follow-up visits [10].

### Patients and outcome variables

All patients with fractures acquired in an e-scooter accident registered in the SFR between 7 April 2019 and 30 December 2022 were included in the study. Data collected included demographics (age and sex), injury date, fracture classification and location, presence of open fracture and primary treatment (non-surgical or surgical). Patients ≥ 18 years old at injury were classified as adults and patients < 18 years as children. Fracture classification was used to group fractures into the following body part locations: clavicle, scapula, humerus, forearm (including distal radius), hand, spine, pelvis, femur, patella, tibia, ankle and foot. Sub-analyses included segment location of long bones: proximal, diaphyseal and distal parts. The primary outcome investigated was the distribution of fractures, expressed as percentage for the respective body part in relation to all fractures. Furthermore, the proportion of surgically-treated fractures and seasonal variation were investigated.

### Ethics, funding, data presentation and potential conflicts of interest

The study was conducted according to the Helsinki Declaration and approval was obtained from the Swedish Ethical Review Authority (Dnr 2022-04355-01). No individual patient consent was needed according to the Swedish Patient Law. The dataset analysed in this study is not freely available because of legislation on register data and restrictions stipulated in the ethical permission only to report aggregated data. Data can be obtained from the Center of Registers, Västra Götaland if ethical approval is granted. The STROBE recommendations for reporting observational studies were applied [11].

The authors declare no competing interests related to this body of work.

### Statistics

Baseline demographic data are presented as the number of patients and fractures, means with standard deviation (SD) and proportions (%). All statistical analyses were performed using R version 4.2.2 [12].

## Results

Some 1,874 fractures in 1,716 patients involved in e-scooter accidents were registered in the SFR and included in the study. The mean age was 29 years and 70% of patients were male. Some 415 fractures (22%) occurred in children. Most were closed fractures (*n* = 1,842, 98%) (Table 1).

**Table 1:** Characteristics of the study population

Patients	1716
Fractures	1874
Mean age (SD)	29 (14)
Sex	
Males	1194 (70%)
Females	522 (30%)
Adult fractures	1459 (78%)
Paediatric fractures (< 18 years)	415 (22%)
Paediatric population’s mean age (SD)	13 years (3)
Open fractures	32 (2%)

### Fracture distribution

**Fig. 1:**
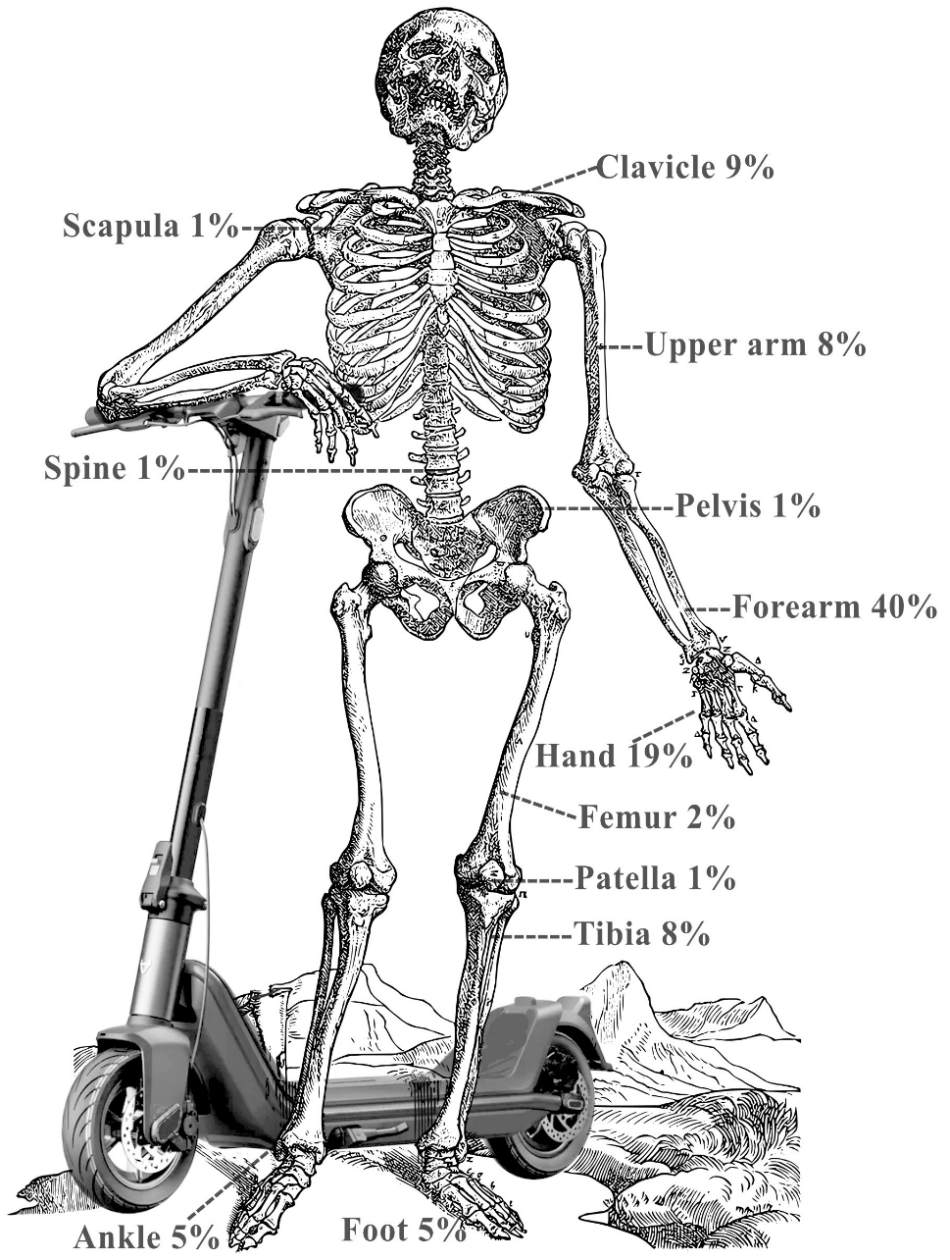
Distribution

The upper extremity was the most prevalent injury site, accounting for 1,459 (78%) fractures. The most frequently injured part of the upper extremity was the forearm (*n* = 757, 40%), followed by the hand (*n* = 363, 20%) (Fig. 1; Table 2).

**Table 2:** Distribution of fractures in e-scooter accidents overall and separately for adults and children, number of fractures (% of group)

Region	Adults (*n* = 1459)	Children (*n* = 415)	Overall (*n* = 1874)	
Clavicle	150 (10.3%)	27 (6.5%)	177 (9.4%)	
Scapula	21 (1.4%)	0	21 (1.1%)	
Upper arm	113 (7.7%)	28 (6.7%)	141 (7.5%)	
Forearm	513 (35.2%)	244 (58.8%)	757 (40.4%)	
Hand	319 (21.9%)	44 (10.6%)	363 (19.4%)	
Spine	7 (0.5%)	2 (0.5%)	9 (0.5%)	
Pelvis	13 (0.9%)	2 (0.5%)	15 (0.8%)	
Femur	31 (2.1%)	6 (1.4%)	37 (2.0%)	
Patella	17 (1.2%)	0	17 (0.9%)	
Tibia	102 (7.0%)	44 (10.6%)	146 (7.8%)	
Ankle	90 (6.2%)	7 (1.7%)	97 (5.2%)	
Foot	83 (5.7%)	11 (2.7%)	94 (5.0%)	

When adults and children were considered separately, the forearm remained the most frequently injured site, with 513 (35% of all adult fractures) adult fractures and 244 (59% of all paediatric fractures) paediatric fractures.

## Surgically treated fractures

Some 556 fractures (30%) were treated surgically. Fractures of the forearm were the leading cause of surgical treatment (*n* = 216 or 39%), with distal radius fractures accounting for most cases (*n* = 114, 21%). This pattern remained when adult (*n* = 455) and paediatric fractures (*n* = 101) were analysed separately, with distal radius fractures being the most frequent injury necessitating surgical treatment in adults (*n* = 78, 17%) and children (*n* = 36, 36%) (Table 3).

**Table 3:** Distribution of fractures treated surgically following e-scooter accidents, long bone fractures presented as proximal, diaphyseal and distal segment fractures. Data are presented overall and separately for adults and children (number (% of surgically treated fractures in respective group)),

Region	Adults (*n* = 455)	Children (*n* = 101)	Overall (*n* = 556)	
**Clavicle**	27 (5.9%)	5 (5.0%)	32 (5.8%)	
**Scapula**	2 (0.4%)	0	2 (0.4%)	
**Humerus** *Proximal* *Diaphyseal* *Distal*	37 (8.1%) 28 (6.2%) 1 (0.2%) 8 (1.8%)	5 (5.0%) 2 (2.0%) 0 3 (3.0%)	42 (7.5%) 30 (5.4%) 1 (0.2%) 11 (2.0%)	
**Forearm** *Proximal* *Diaphyseal* *Distal radius*	162 (35.6%) 62 (13.6%) 22 (4.8%) 78 (17.1%)	54 (53.4%) 2 (2.0%) 16 (15.8%) 36 (35.6%)	216 (38.8%) 64 (11.5%) 38 (6.8%) 114 (20.5%)	
**Hand**	50 (11.0%)	6 (5.9%)	56 (10.1%)	
**Spine**	0	0	0	
**Pelvis**	1 (0.2%)	0	1 (0.2%)	
**Femur** *Proximal* *Diaphyseal* *Distal*	30 (6.6%) 24 (5.3%) 3 (0.7%) 3 (0.7%)	4 (4.0%) 2 (2.0%) 1 (1.0%) 1 (1.0%)	34 (6.1%) 26 (4.7%) 4 (0.7%) 4 (0.7%)	
**Patella**	5 (1.1%)	0	5 (0.9%)	
**Tibia** *Proximal* *Diaphyseal* *Distal*	70 (15.4%) 50 15 5	21 (20.7%) 3 (2.9%) 6 (5.9%) 12 (11.9%)	91 (16.4%) 53 (9.5%) 21 (3.8%) 17 (3.1%)	
**Ankle**	57 (12.5%)	6 (5.9%)	63 (11.3%)	
**Foot**	14 (3.1%)	0	14 (2.5%)	

### Seasonal and weekly variation of injuries

**Fig. 2:**
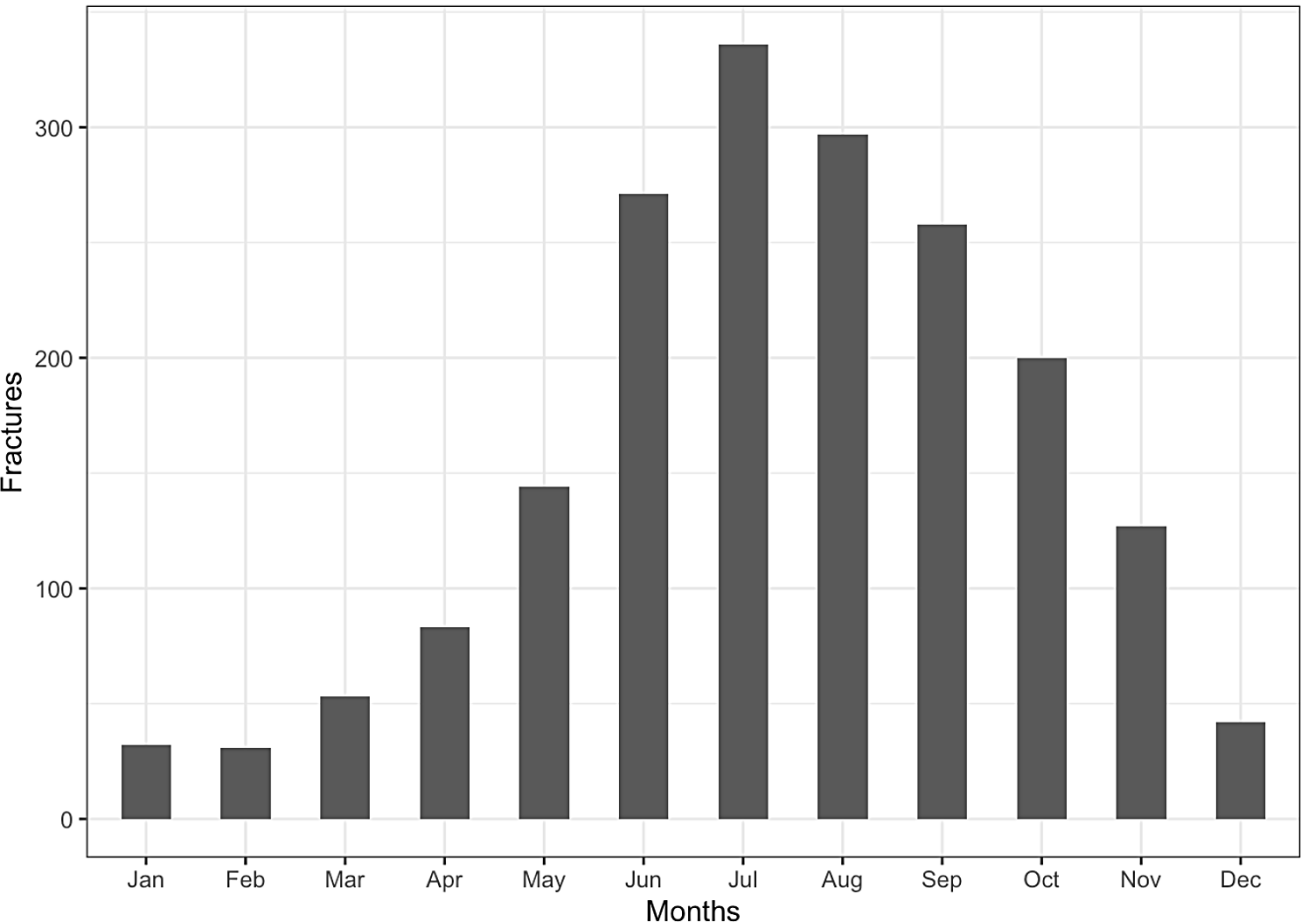
Months

Our analysis of monthly variation indicates a prominent spike in e-scooter-related fractures during the summer months, with July having the highest number of accidents (Fig. 2).

**Fig. 3:**
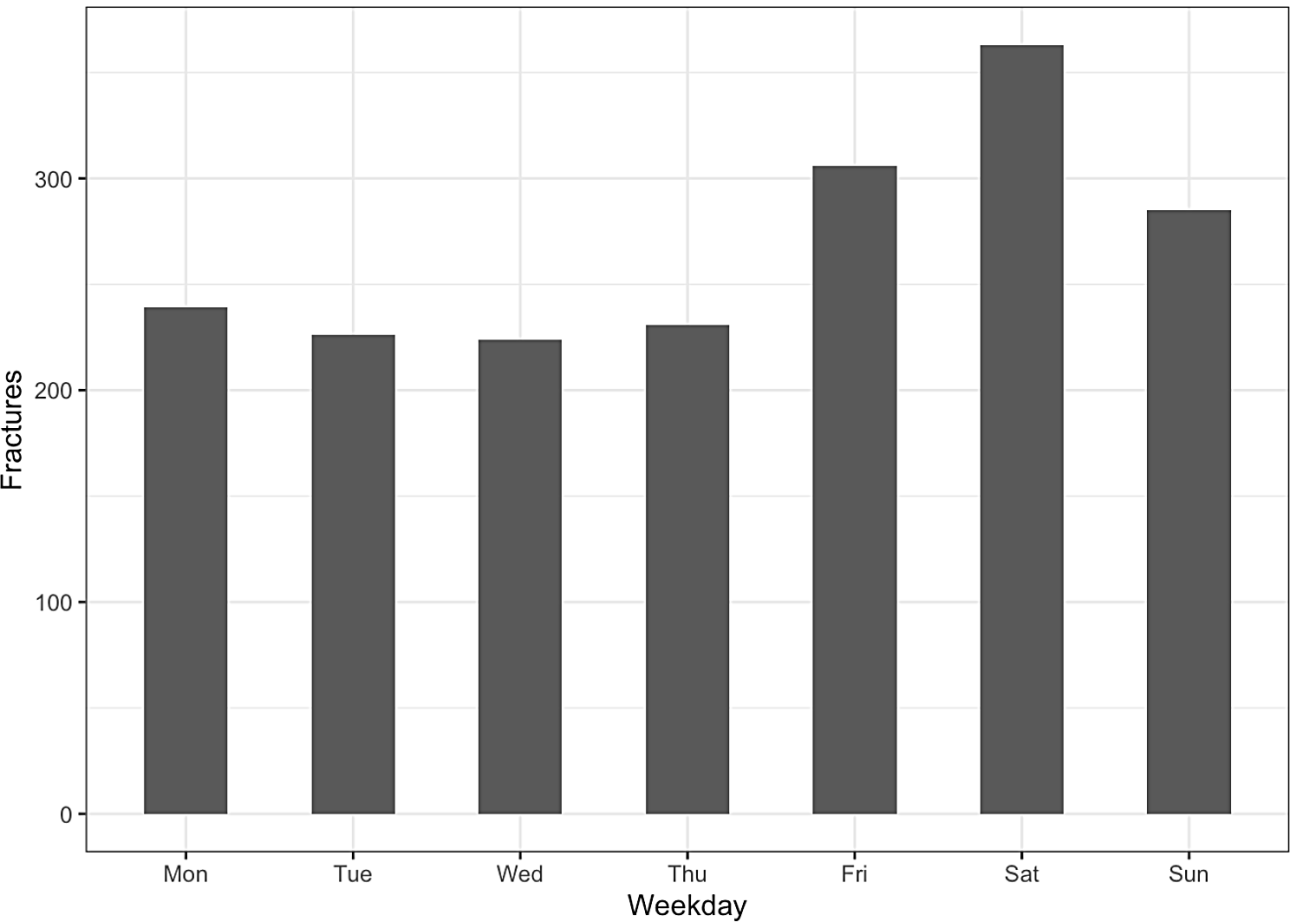
Days

Regarding weekly variation, Saturdays had the highest occurrence of e-scooter-related fractures, contributing to the overall higher injury rate on weekends (Fig. 3).

## Discussion

During the study period, e-scooter accidents resulted in 1,874 registered fractures. The majority of these fractures occurred in the upper extremity, particularly the forearm.

This pattern did not change when adults and children were analysed separately. Most patients in our study were younger males (mean age 29 years). These findings are in line with those reported by a recent systematic review of 34 studies examining injuries after e-scooter accidents [13]. The mean age of the participants in that review was 33 years and 58% (vs. 70% in our study) were males. The most common type of injury was bony injuries in which upper extremity fractures dominated. Unlike our study, that review encompassed studies investigating non-orthopaedic injuries such as head injuries, lacerations and contusions.

James et al. estimated a mortality rate of 9% in road traffic accidents with e-scooters, highlighting that injuries caused by e-scooters transcend orthopaedic diagnoses [14].

Other studies focusing on orthopaedic injuries corroborate our findings, where sex and age distributions align with previous studies investigating e-scooter-associated injuries [14–17].

The main focus of the current study was to assess the distribution of orthopaedic injuries caused by e-scooter accidents, rather than the associated burden. Nevertheless, given that 1,874 fractures were registered during the study period of roughly 3,5 years, out of which one third required surgical treatment, these injuries alone constitute a significant burden on healthcare systems especially given that the true number of injuries is likely significantly higher.

Ahluwalia et al. [18] found that injuries caused by e-scooter accidents impose a substantial burden on the healthcare system. The authors describe an average cost of almost 1,500 £ per patient in their study of 202 e-scooter injuries. Another study assessed the absence of work secondary to e-scooter accidents and found - quite surprisingly - that non-surgically treated patients were absent for about 1 month and surgically treated patients for up to 3 months [19].

A rise in e-scooter related injuries during the summer months was observed, consistent with previous research [15, 20]. This variation is not surprising because summer is the peak time for e-scooter usage, leading to increased demand. E-scooter use is not ideal in Sweden during winter due to snowy and icy road conditions. A Swedish study on e-scooter injuries [15] also found a peak in injuries during the weekend. Intoxication could be the reason behind the increase in weekend injuries seen in our studies, as studies have shown a strong link between intoxication and e-scooter injuries [21–23]. Because we lack data on alcohol and drug consumption, this contention is purely speculative.

By identifying common fractures caused by e-scooter accidents, we hope to raise awareness about the importance of safe e-scooter usage, including wearing protective gear, encouraging injury prevention measures and ultimately reducing the incidence and severity of orthopaedic injuries in this growing population.

### Strengths and limitations

The major strength of the study is the large number of fractures that ensured an adequately large sample size to calculate the distribution of fractures caused by e-scooter accidents reliably. The SFR is a reliable source of information regarding demographic data, fracture classification and choice of treatment. However, because the analysis is based on the SFR, only injuries resulting in a fracture contingent on orthopaedic treatment are included. Hence, other injuries that may arise in e-scooter accidents, such as head injuries, thoracic and abdominal injuries and lacerations, are not reported. Fatally injured patients will never be included in the SFR. Moreover, the SFR, in its present form, does not report injuries with e-scooter involved suffered by pedestrians or cyclists as e-scooter injuries. Combined, these factors add to an underestimation regarding true impact of e-scooter-related fractures. Our study did not aim to describe the overall incidence of e-scooter accidents, but rather focused on the distribution of e-scooter orthopaedic injuries and their treatment.

As in all register-based studies, our study runs the risk of miscoding, misclassification, and transferring errors.

#### Future perspectives

With the help of data from the SFR, future investigations can examine trends in e-scooter-related orthopaedic trauma. A more comprehensive depiction of the orthopaedic trauma caused by e-scooter injuries could be achieved by adjusting the SFR to account for other injuries (e.g., individuals hit by an e-scooter or who trip over one). In addition, enabling registration of skull and rib fractures would strengthen the register’s ability to make an accurate description of the full panorama of skeletal injuries resulting from e-scooter accidents.

## Conclusion

E-scooter-associated orthopaedic injuries tend to primarily affect the upper extremity. They constitute an increasing burden on emergency and orthopaedic departments in Sweden and other countries. The majority of patients were younger males, and there was a peak in injuries during summer and weekends. The study highlights the need for implementation of protective measures, in order to minimize these injuries.

## Data Availability

This study is based on data from the Swedish Fracture Register, who can be contacted via frakturregistret@vgregion.se.
